# Trajectories of Mother-Infant Communication: An Experiential Measure of the Impacts of Early Life Adversity

**DOI:** 10.3389/fnhum.2021.632702

**Published:** 2021-02-17

**Authors:** Lauren Granata, Alissa Valentine, Jason L. Hirsch, Jennifer Honeycutt, Heather Brenhouse

**Affiliations:** ^1^Department of Psychology, Northeastern University, Boston, MA, United States; ^2^Department of Psychology, Bowdoin College, Brunswick, ME, United States

**Keywords:** early life adversity, maternal separation, limited bedding, development, ultrasonic vocalizations, rat

## Abstract

Caretaking stability in the early life environment supports neurobehavioral development, while instability and neglect constitute adverse environments that can alter maturational processes. Research in humans suggests that different types of early life adversity (ELA) can have differential effects on caretaker relationships and later cognitive and social development; however, identifying mechanistic underpinnings will require animal models with translational validity. Two common rodent models, maternal separation (MS) and limited bedding (LB), influence the mother-infant relationship during a critical window of development. We hypothesized that these paradigms may affect the development of communication strategies on the part of the pup. Ultrasonic vocalizations (USVs) are a care-eliciting mechanism and ethologically relevant response to stressors in the rat pup. USV emission rates and acoustic parameters change throughout early development, presenting the opportunity to define developmental milestones in USVs that would reflect neurobehavioral aberrations if disrupted. This study investigated the effects of MS or LB on the dam-pup relationship by quantifying pup USVs, maternal behavior, and the relationship between the two. First, we used a generalized additive model approach to establish typical developmental trajectories of USV acoustic properties and determine windows of change in MS or LB rearing. Additionally, we quantified maternal behaviors and the predictability of maternal care sequences using an entropy rate calculation. MS and LB each shifted the developmental trajectories of USV acoustic parameters and call types in a sex-specific manner. MS more often impacted male USVs, while LB impacted female USVs. MS dams spent more time passive nursing, and LB dams spent more time on the nest. The predictability of maternal care was associated with the rate of USV emissions exclusively in females. Taken together, findings demonstrate sex- and model-specific effects of rearing environments on a novel developmental trajectory involving the mother-infant relationship, facilitating the translation of animal ELA paradigms to assess later-life consequences.

## Introduction

The nature of the parent-offspring relationship is a particularly critical component of the early postnatal environment in mammals. In humans, quality of parental care has been shown to be predictive of adolescent and adult psychopathologies, contributing to variations in stress and anxiety regulation (McGoron et al., 2012; Kundakovic and Champagne, 2015). For example, parental abuse and neglect, including cases of severe deprivation in institutionalized infants, is associated with increased rates of autistic-like traits, personality and anxiety disorders, and depression (Trickett and McBride-Chang, 1995; Johnson et al., 1999; Rutter et al., 1999; Enoch, 2011). Unsurprisingly, the long-term outcomes of early life adversity (ELA) have been widely studied in humans, non-human primates, and rodents. Such research corroborates that the neonatal window defines a critical period wherein experiences shape developmental trajectories.

As clinical observations dating back to Erikson (1959) have shown, developmental milestones marking critical periods for affective and cognitive maturation during early life can predict later function. An example of a specific predictor of later cognitive or emotion-regulation deficits include failure to meet language development milestones by 24 months (Peyre et al., 2017). Since the timing and sequential pattern of milestone acquisition form an important marker of neurological integrity, these measures can provide crucial information regarding individualistic disturbances attributable to early adversity. Traditionally, animal models have allowed the investigation of mechanisms underpinning effects of adversity on later-life behaviors, proving to be useful when parsing effects of specific experiential domains on development. However, to date, the evidence associating discrete facets of ELA with developmental milestone achievement in the context of socio-emotional development are lacking.

Caretaker behavior is crucial to mammalian development, regulating physiological homeostasis and suppressing the adrenocortical response during the preweaning period (Sapolsky and Meaney, 1986; Hofer, 1996; Champagne et al., 2001; Zimmerberg et al., 2003). Thus, rodent models of ELA typically employ methods to disrupt dam-infant interactions, leading to long-term effects in offspring. In one such model, maternal separation (MS), pups are separated from the dam for a specified time daily during the preweaning period. MS results in changes in the hypothalamic-pituitary adrenal (HPA)-axis stress response, immune function, neuronal morphology, and functional connectivity in the prefrontal cortex and connected regions (Lehmann et al., 2002; Aisa et al., 2007; Enoch, 2011; Chocyk et al., 2013; Wieck et al., 2013; Holland et al., 2014). These changes occur alongside behavioral aberrations such as increased anxiety-like behavior and social deficits (Monroy et al., 2010; Holland et al., 2014; Ganguly et al., 2015). While MS inherently reduces the amount of time the pups spend in the dam’s presence, there is also evidence that dams engage in compensatory care after being reunited with pups (Huot et al., 2004). Partially to limit this compensatory behavior and interactions with experimenters, the limited bedding and nesting model (LB) was developed. LB disturbs normal rearing by providing inadequate nesting material in the cage, which, in turn, influences maternal behavior (Gilles et al., 1996; Brunson et al., 2005; Walker et al., 2017). In this paradigm, the dam’s basal corticosterone levels increase, leading to fragmented, unpredictable, and abusive care patterns (Molet et al., 2016; Walker et al., 2017; Gallo et al., 2019). Pups reared under LB conditions likewise show deviations in HPA axis regulation in addition to later-life neuronal dysfunction, anhedonia, cognitive deficits, and anxiety-like behavior (Brunson et al., 2005; Bath et al., 2016; Molet et al., 2016).

Data from both the human and animal literature have found certain outcomes associated with ELA to be sex-specific (MacMillan et al., 2001; Dickie and Armony, 2008; Bath et al., 2016; Grassi-Oliveira et al., 2016; Bondar et al., 2018). In rodents, this effect may be partially attributable to male and female pups receiving different levels of caregiving behaviors. For example, dams preferentially direct anogenital licking to male pups (Richmond and Sachs, 1983). Variations in this behavior regulate epigenetic modifications contributing to sexually dimorphic development of the stress and reproductive systems (Champagne and Meaney, 2001; Renard et al., 2007; Roth et al., 2009). The specific factors of adverse rearing environments that mediate sex-dependent pup-directed behaviors have not been widely explored.

Dam-pup interactions are facilitated in part by auditory signals in the form of isolation-induced ultrasonic vocalizations (USVs) (Muller et al., 2010). Recent research supports that USVs are an indicator of affective state and elicit maternal care behaviors from the dam, not simply a byproduct of general arousal (Muller et al., 2010). Thus, from an evolutionary standpoint, vocalizations are critical for pup survival (Branchi et al., 2001; Ehret, 2005; Wöhr and Schwarting, 2008; Lingle et al., 2012). USVs undergo developmental changes during the preweaning period, with call duration, peak frequency, and bandwidth increasing from P10 to P17 (Brudzynski et al., 1999). Further, male pups emit USVs at a greater rate and a lower frequency than females (Bowers et al., 2013), but developmental trajectories for USVs have not been clearly defined in both sexes. If these acoustic parameters contribute to sex-specific caregiving patterns and carry functional significance in neurobehavioral development, USVs in growing pups could be interpreted as developmental milestones, where shifts or delays indicate atypical development (Grimsley et al., 2011). Standard milestones of early development include physical and reflexive measures such as eye opening, fur development, and the mid-air righting reflex, which typically appear between P10 and P20 (Heyser, 2004). Notably, these physical milestones are impacted by both MS and LB in a sex-dependent manner (Demaestri et al., 2020). Including an ethologically relevant measure of affective state, such as infant USVs, will contribute to the translational validity of rodent models of ELA.

The current experiments sought to define developmental trajectories for acoustic parameters of USVs in male and female rats in order to assess the pups’ affective response to adversity over time. Firstly, we built a model of the typical development of USV parameters. These include standard acoustic measures in addition to analysis by the USV’s sonographic structure or call type, which have been hypothesized to play a role in dam-pup communication (Brudzynski, 2005). The MS and LB models of ELA were implemented to determine their individual effects on the model of typical USV development. We assessed each paradigm’s effects on the model USV curves to identify critical periods when vocalizations were susceptible to environmental changes. The second goal of the study was to determine how rearing condition influenced maternal behaviors. Frequency and duration of tactile stimulation, via grooming and engaged nursing is known to support pup development (Champagne et al., 2001). In addition, there is growing evidence of the importance of the temporal predictability of maternal care behavior in regulating neurobehavioral outcomes (Walker et al., 2017). Thus, we assessed individual rat dam behaviors and the predictability of behavioral sequences by calculating the entropy of behavioral transitions. Finally, to investigate the degree to which maternal care was associated with changes in the pups’ characteristics, we assessed whether maternal behaviors covaried with differences in USV emissions between rearing conditions. These studies were aimed to elucidate individual differences in dam-pup interactions that contribute to the dysfunctional behavioral development in two widely used models of ELA.

## Materials and Methods

### Subjects

**FIGURE 1:**
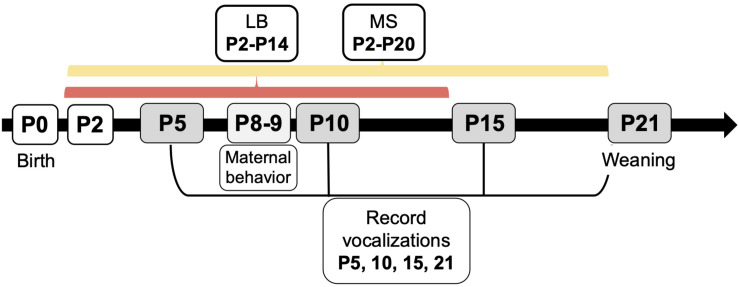
Experimental timeline for early life stress paradigms, ultrasonic vocalization recordings, and maternal behavior recordings. Whole litters were assigned to control (Con), maternal separation (MS), or limited bedding (LB) groups at birth. On P2, MS litters began daily separations from dam and littermates until P20, and LB litters began housing in standard cages with a wire mesh floor and one-half of a paper towel provided for nesting material until P14. Con litters were reared in standard conditions until weaning. Ultrasonic vocalizations (USVs) were recorded on P5, 10, 15, and 21. Maternal behavior was monitored from P8-P9.

All experiments were performed in accordance with the 1996 Guide for the Care and Use of Laboratory Animals (NIH) with approval from the Institutional Animal Care and Use Committee at Northeastern University. Male and female Sprague-Dawley rats originally obtained from Charles River Laboratories (Wilmington, MA) were used to breed subjects for this study. One male and one female were caged together until pregnancy was confirmed, a maximum of 4 days. All females were primiparous, and pregnant dams were housed singly. Parturition was checked daily, and the day of birth was denoted as P0. On P1, litters were culled to 10 pups, maintaining 5 males and 5 females wherever possible. Whole litters were randomly assigned to be reared under control (Con), MS, or LB paradigm. To avoid litter effects, a maximum of two males and two females were used from each litter (*n* = 10/group). Dams and pups in Con rearing were housed under standard laboratory conditions in polycarbonate wire-top caged with a 2–3 inch layer of pine shave bedding covering the bottom of the cage. Food and water were available *ad libitum*. The facility was kept on a 12-h light/dark cycle (light period between 0700 and 1900) with regulated temperature (22–23°C) and humidity (37–53%). Con pups were left undisturbed except to be weighed on P9 and P20, and USV recording days on P5, P10, P15, and P21 (Figure 1). Pups were identified by toe clips performed on P5 after USVs were recorded.

### Maternal Separation

From P2-20, MS pups were separated from their dams and littermates for 4 hours per day (0900–1300 h) as previously described (Holland et al., 2014; Grassi-Oliveira et al., 2016; Ganguly et al., 2019). MS dams remained in their home cage in a separate room. From P2-10, separations took place in individual plastic cups with home cage pine shavings and droppings in a circulating water bath kept to 37°C to maintain nest odor and temperature. From P11-20, when pups are able to thermoregulate, they were individually separated in small mouse cages with the same home cage pine shavings. Pups were returned to dams in standard laboratory cage conditions identical to Con.

### Limited Bedding

On P2, LB dams and litters were transferred to a cage with thin layer of pine shaving bedding placed under a wire grid (0.25″× 0.25″ grid size) resting 0.5″ off the cage floor (Molet et al., 2014; Walker et al., 2017). A small handful of bedding and half of a C-fold paper towel were placed on top of the wire mesh for nesting material. This arrangement allows waste to fall beneath the wire grid without contributing to nesting material in the cage. LB housing took place until P14. Therefore, LB pups experienced a different total amount of time in the adversity rearing condition than MS pups. These timelines were chosen because of their standardization as accepted timelines in previous literature. Additionally, the purpose of the study was not to make direct comparisons between the two paradigms, but to examine the responses compared to the control group. For this study, MS and LB pups may be considered to have received the same “dose” of adversity for all outcome measures with the exception of USVs on P15 and P21.

### USV Recording and Classification

USVs were recorded for the same pup (identified by toe clips) on P5, 10, 15, and 21. Pups from each rearing condition were recorded in the same manner. Each pup was isolated for 5 min while audio was recorded with an ultrasonic microphone (Avisoft Bioacoustics, model CM16/CMPA) positioned 10 cm above the cage. On P5 and 10, pups were recorded in individual plastic cups with home cage pine shavings in the water bath kept at 37°C. Since cold temperatures and newly changed bedding have been shown to induce USVs, pups were kept at a thermoneutral temperature to obtain a naturalistic simulation of isolation in a familiar environment. On P15 and P21, pups were recorded in small mouse cages with home cage bedding. Following the recording, the pup was returned to the home cage.

**FIGURE 2:**
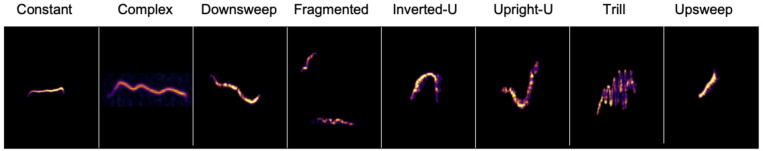
Representative spectrograms of USV call types.

Audio files were uploaded and analyzed using Deepsqueak (Coffey et al., 2019), a Matlab program for USV analysis. USVs were visualized by converting each file to spectrograms using Fast Fourier Transformation (FFT). Each call detected by Deepsqueak was manually confirmed or rejected by a trained experimenter. Deepsqueak uses contour detection to generate call statistics from the spectrograms of accepted calls and accurately separates USVs from broad spectrum noise. The duration, maximum frequency, minimum frequency, and peak frequency of each call are extracted from the contour. To cluster USVs by sonographic structure using the shape, frequency, and duration from the contours, a previous dataset of 12,227 USVs from 75 subjects was used to train a supervised classifier in Deepsqueak. USV types were developed based on pup calls identified by Brudzynski et al. (1999). The types identified in manual classification of the training dataset were complex, constant, downsweep, fragmented, inverted-U or upright-U, upsweep, and trill (call type criteria outlined in Table 1; representative images in Figure 2). In the supervised classification, trills and complex calls were combined into one category due to high confusion matrix correlations between the two types.

**TABLE 1:** USV call type descriptions for manually classified calls that were used to train the supervised classifier.

Call Type	Criteria
Complex	At least 3 changes in frequency sweeps
Constant	0 changes in frequency sweeps
Downsweep	Continuous downward slope
Fragmented	At least 1 frequency jump fluid in time
Inverted-U or Upright-U	1 frequency change Upward to downward or downward to upward
Trill	Rapid frequency fluctuations over large bandwidth (>3 changes in frequency; bandwidth > 8 kHz)
Upsweep	Continuous upward slope

### Maternal Behavior Assessment

Beginning on P8, home cage recordings were taken at three 30-min time-points (at 1430 and 2330 on P8 and 0830 on P9) for later assessment of maternal behavior. A red light was used during the dark phase recording (2330). One time point during the dark phase (2330) was chosen to account for changes in maternal behavior due to nocturnality (Capone et al., 2005). An experimenter blind to experimental condition coded the videos continuously using EthoVision’s manual scoring feature to acquire total durations the dam engaged in each behavior assessed. Dams were identified as engaging in one of the following behavior states: on nest, off nest, arched-back nursing (ABN), passive nursing, licking and grooming (LG), licking and grooming while nursing, and self-grooming (Capone et al., 2005). The dam was considered on the nest when she was in the same cage quadrant as the nest. ABN was scored if the dam was positioned over the nest with legs in a wide stance and an arch or curved back that is repeatedly initiated. Passive nursing was scored when pups were latched and nursing, but the dam is unengaged, sleeping, or laying down. LG was scored when the dam licked one or more pups. LG occurring during a nursing bout was scored separately. We added LG with and without nursing for the final calculation of LG duration. Self-grooming was scored when the dam licked herself or cleaned her face. Scoring reliability of above Cohen’s kappa = 0.9 was established between two experimenters using Cohen’s kappa reliability testing.

### Statistical Analyses

#### USVs

Statistics were calculated in R 3.6.2 (R Core Team, 2019). Generalized additive models (GAMs) were used to assess the effects of age on USV parameters. GAMs are predictive models that incorporate a smooth function of one or more covariates to model non-linear relationships without assuming the shape of the model *a priori*. The *mcgv* (Wood, 2003) package was used to specify the model according to the following form:

$$y_{ij}=\alpha_{0}+\alpha_{1}Rearing_{ij}+f_{j}\left(Age_{i}\right)+\varepsilon_{i}$$

where α_0_ is the intercept, α_1_ is the difference between the mean response in the j^*th*^ rearing condition and α_0_, f_*j*_(Age) is the smooth function representing the relationship with age for each rearing condition, and ε_*i*_ is the residual (Rose et al., 2012). The smooth function was constructed with four knots and a “by” variable, which allows the smoothing of age to interact with rearing condition as a factor and thus incorporates separate age-dependent response curves for each rearing condition into a single model. Periods of age-dependent change were identified using the gratia (Simpson and Singmann, 2020) package to perform a posterior simulation of 10,000 iterations and calculate the first derivative of their estimated smooths using finite differences. Derivatives and their 95% confidence intervals were computed from a multivariate normal distribution fitted to the model parameters. Ages where confidence intervals did not include zero (*p* < 0.05) were identified as periods of age-related change.

In the first set of analyses, GAMs were performed for males and females separately. All rearing conditions were included in the model, but MS and LB were independently compared to Con. To determine age periods where MS or LB models differed from Con, prediction matrices of fitted values of the responses were created with the GAM as specified above. Then, rows of the new matrix corresponding to MS or LB were subtracted from those corresponding to Con, and standard errors were calculated to generate 95% confidence intervals of the difference between rearing conditions (Rose et al., 2012). Significant differences between Con and MS or LB were identified as ages where 95% confidence intervals did not include zero (*p* < 0.05). A second analysis was performed to determine the effects of sex within each rearing condition. We determined age-related changes using a GAM defined by the following model:

$$y_{ij}=\alpha_{0}+\alpha_{1}Sex_{ij}+f_{j}\left(Age_{i}\right)+\varepsilon_{i}$$

Differences between males and females were determined using the methods described above.

#### Maternal Behavior Group Comparisons

Maternal behavior on P8-9 was directly compared between paradigms, as at this time the differing length of adversity paradigms was not relevant. Maternal behavior analyses were conducted using the *rstatix* (Kassambara, 2020) package in R. Data were assessed for normality of residuals using the Shapiro-Wilk Test and homogeneity of variances using Levene’s Test. One-way ANOVAs were conducted to compare the total durations and frequencies of maternal behaviors between rearing groups at each time point. Significant ANOVAs were followed by Tukey’s HSD *post-hoc* multiple comparisons tests to determine differences between each rearing condition. One Con litter and three MS litters were excluded from analysis at the 2330 time point due to technical failure of the red light, making videos unviewable. Results were visualized using GraphPad Prism 7.

#### Entropy Analysis

Entropy scores were calculated for each dam’s individual transition matrix according to the formula:

$$H\left(\chi \right)=-\sum_{ij}\mu_{i}P_{ij}\log P_{ij}$$

where P_*ij*_ is the probability of transitioning from behavior i to behavior j, and m_*i*_ is the stationary distribution. Because some probability matrices were singular and thus non-invertible, the stationary distribution was estimated with the generalized inverse (*ginv*) function in R.

Analyses of covariance (ANCOVA) were calculated to test for any covariance of pup weight with maternal entropy scores (at P8-9) or total number of USVs (at P21). ANCOVA were also calculated to determine the effect of rearing on the total number of USVs emitted at each age while adjusting for maternal entropy scores at each time point analyzed. Results were evaluated for statistical significance using a bonferroni-corrected *p*-value of 0.0127 to account for multiple comparisons. All statistics were carried out in R using the *rstatix* (Kassambara, 2020), *emmeans* (Lenth et al., 2020), and *lsr* (Navarro, 2015) packages to assess normality of residuals using Shapiro-Wilk Test, homogeneity of variances using Levene’s Test, and effect sizes (h^2^), and results were visualized using *ggplot2* (Wickham et al., 2020). Observations with standardized residuals greater than 3 were identified as outliers and removed from analysis.

## Results

### USV Acoustic Properties

Statistics for USV acoustic properties (total calls, average peak frequency, and average bandwidth) compared between rearing conditions within sex can be found in Table 2. Statistics for USVs by call type can be found in Table 3. In order to reveal sex effects, statistics directly comparing male and female USV acoustic properties within each rearing condition can be found in Supplementary Table 1.

**TABLE 2:** Summary statistics of USV acoustic properties analyzed by generalized additive models comparing rearing conditions within each sex.

			edf	Ref.df	F	*p*-value	Periods of age-related changes	Age periods significantly different from Con
Total USVs (#)	Males	Con	2.141	2.511	3.459	0.01889*	5–11.27 (+)	
		MS	2.715	2.936	10.264	3.10E-05***	5–9.82 (+) 12.72–21 (−)	6.45–12.16 (>Con) 17.3–21 (<Con)
		LB	2.787	2.964	4.778	2.63E-03**	8.86–15.05 (+) 18.27–21 (−)	
	Females	Con	1.993	2.364	2.834	0.0543		
		MS	2.051	2.423	3.754	0.0197*	14.89–21 (−)	
		LB	2.92	2.995	9.74	5.58E-06***	8.78–15.21 (+) 16.34–21 (−)	6.21–10.39 (<Con) 13.36–19.31 (>Con)
Average peak frequency (kHz)	Males	Con	1.983	2.358	13.79	0.00000138***	5–15.37 (+)	
		MS	2.916	2.994	21.43	1.66E-11***	8.54–16.26 (+) 18.99–21 (−)	6.61–11.27 (<Con) 14.49–19.47 (>Con)
		LB	1	1	12.33	0.000645***	5–21 (+)	12.24–16.58 (<Con)
	Females	Con	1.598	1.937	5.525	0.00385**	5–14.89 (+)	
		MS	2.395	2.736	5.693	1.12E-03**	6.53–13.04 (+)	
		LB	2.59	2.872	4.147	0.02148*	5–9.9 (+)	8.46–11.43 (>Con)
Average bandwidth (kHz)	Males	Con	2.406	2.744	6.609	0.002122**	5–10.47 (+) 15.21–17.54 (−)	
		MS	2.479	2.798	7.678	7.41E-04***	5–10.07 (+) 14.17–21 (−)	
		LB	2.256	2.62	8.652	0.000104***	5–11.75 (+) 16.18–21 (−)	
	Females	Con	2.171	2.539	5.909	0.00193**	5–11.19 (+) 16.18–21 (−)	
		MS	2.732	2.944	6.2	2.44E-03**	5–9.82 (+) 13.36–17.14 (−)	
		LB	2.831	2.977	11.848	4.63E-07***	7.17–14.57 (+) 16.1–21 (−)	6.21–8.38 (<Con) 13.36–18.91 (>Con)

Effective degrees of freedom (edf) indicates the degree of non-linearity of the age-outcome relationship (edf = 1 for linear relationships). Periods of age-related change based on derivates of GAM models are denoted by (+) for increases and (−) for decreases. Significant differences between MS or LB and Con are based on differences in GAM predictions and 95% confidence intervals. *p < 0.05, **p < 0.01, ***p < 0.001. n = 9–10.

**TABLE 3:** Summary statistics of USV call types as a percentage of total calls emitted.

			edf	*F*	*p*	Periods of age-related changes	Age periods significantly different from Con
**Complex (%)**	Males	Con	1.027	4.136	0.045*	9.66–19.87 (+)	
		MS	1.671	0.934	3.99E-01		5–6.13 (>Con)
		LB	1.663	0.819	0.437		
	Females	Con	1	6.433	0.0125*	5–21 (+)	
		MS	1.469	0.527	6.39E-01		
		LB	1.609	0.969	0.3219		5–8.14 (>Con) 14.73–20.84 (<Con)
Constant (%)	Males	Con	2.654	15.226	0.0000001***	5–10.87 (−)	
		MS	2.115	8.302	2.87E-04***	5–11.43 (−)	
		LB	2.634	16.942	1.97E-08***	5–11.19 (−)	
	Females	Con	2.437	17.266	2.53E-08***	5–11.51 (−)	
		MS	2.199	7.085	0.000928***	5–11.11 (−)	
		LB	2.106	11.647	8.57E-06***	5–12.56 (−)	
**Downsweeps (%)**	Males	Con	1.025	8.575	0.00351**	5–21 (−)	
		MS	1.996	1.407	1.88E-01**	9.42–10.31 (−)	20.52–21 (>Con)
		LB	2.789	2.535	0.08201		
	Females	Con	1	0.965	0.3281		
		MS	1	0.175	6.76E-01		
		LB	2.797	2.577	0.0722		
**Fragmented (%)**	Males	Con	2.498	3.516	0.05533		
		MS	2.87	12.032	2.19E-06***	5–9.66 (+) 11.19–18.19 (−)	7.41–11.99 (>Con) 18.59–20.2 (<Con)
		LB	2.205	6.389	8.26E-04***	5–12.16 (+)	5.96–8.3 (<Con) 16.34–19.47 (>Con)
	Females	Con	1.686	0.844	0.451725		
		MS	2.856	6.804	0.000667***	5–9.34 (+) 11.03–16.9 (−)	7.01–12.48 (>Con) 17.46–19.87 (<Con)
		LB	2.878	9.77	5.59E-06***	8.46–15.21 (+) 16.74–21 (−)	5–9.98 (<Con) 12.8–19.95 (>Con)
**Inverted-U or Upright-U (%)**	Males	Con	1	0.751	0.388		
		MS	1	2.274	1.34E-01		
		LB	1	0.815	0.369		
	Females	Con	1	0.435	0.5111		
		MS	1.743	1.634	1.87E-01		
		LB	2.337	2.814	0.0842		
**Upsweeps (%)**	Males	Con	2.143	4.489	0.00622**	6.53–12.4 (+)	
		MS	2.747	10.151	5.64E-06***	8.7–16.18 (+)	7.17–9.9 (<Con) 18.27–19.79 (>Con)
		LB	1	7.386	0.00763**	5–21 (+)	12.32–14.73 (<Con)
	Females	Con	1.822	3.032	0.04538*	6.29–10.95 (+)	
		MS	1.576	5.804	3.15E-03**	5.88–15.53 (+)	
		LB	1.135	2.281	0.10153		

GAMs were performed for males and females separately, comparing MS or LB to Con. Effective degrees of freedom (edf) indicates the degree of non-linearity of the age-outcome relationship (edf = 1 for linear relationships). Periods of age-related change based on derivates of GAM models are denoted by (+) for increases and (−) for decreases. Significant differences between MS or LB and Con are based on differences in GAM predictions and 95% confidence intervals. Bolded call types indicate an effect of both age and rearing. *p < 0.05, **p < 0.01, ***p < 0.001. n = 9–10.

#### Total USVs

To ensure that total USV rate was not attributable to variability in pup weight, analyses of covariance (ANCOVAs) were conducted for P20 total USV number with pup weight at P20 as a covariate. Pup weight was not found to covary with USV emissions (*p* > 0.05 for both males and females; Supplementary Table 2).

**FIGURE 3:**
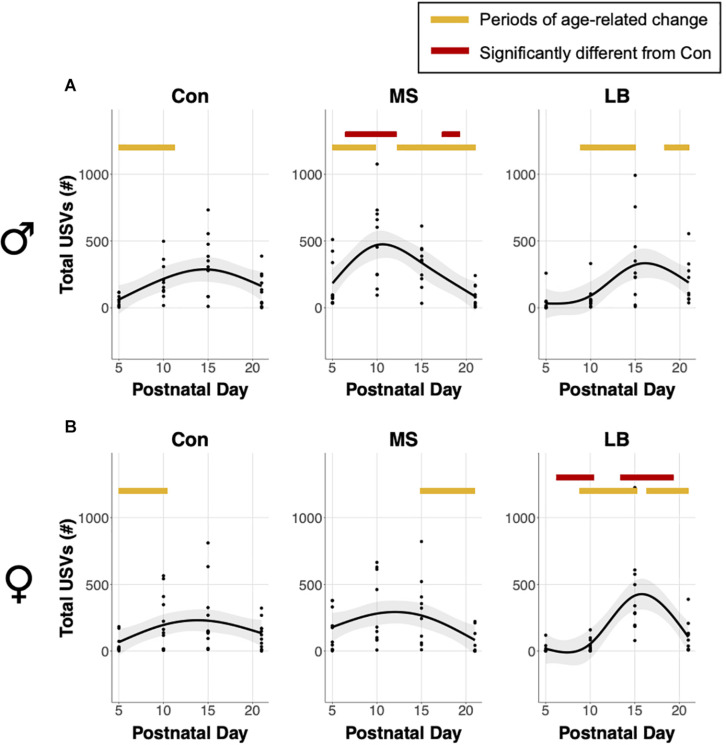
Effects of age and maternal separation (MS) or limited bedding (LB) on total ultrasonic vocalizations (USVs) emitted in **(A)** males and **(B)** females. Shaded areas represent 95% confidence intervals. Orange bars indicate periods of significant age-related change based on the derivative of the GAM model fit. Red bars indicate periods significantly different from control (Con) based on differences between GAM models and confidence intervals. *n* = 9–10.

Analysis of generalized additive model fits of total USV emissions indicate that in males, all rearing groups follow a developmental trajectory dependent on age (approximate significance of smooth terms (SST): Con: *F* = 3.459, *p* = 0.019^∗^; MS: *F* = 10.264, *p* < 0.001^∗∗∗^; LB: *F* = 4.778, *p* = 0.003^∗∗^; Figure 3A). 95% confidence intervals of model derivatives identified periods of significant age-related change in Con (increasing P5-11.27), MS (increasing P5-9.82 and decreasing P12.72-21), and LB (increasing P8.86-15.05 and decreasing P18.17-21). Differences between the prediction curves demonstrated that MS pups emitted more calls than Con between P6.45 and P12.16, but fewer calls between P17.3 and P21.

In females, MS and LB groups demonstrated significant age-related change in total USV number, while Con animals demonstrated a trending, but non-significant age-related change (SST: Con: *F* = 2.834, *p* = 0.0543; MS: *F* = 3.754, *p* = 0.0197^∗^; LB: *F* = 9.74, *p* < 0.001^∗∗∗^; Figure 3B). The model predicts a decrease in USVs in MS females from P14.89-21. LB USVs increased between P8.78 and P15.21, and subsequently decreased between P16.34 and P21. The model predicts that LB emitted fewer calls than Con between P6.21 and P10.39, and more calls than Con between P13.36 and P19.31.

#### Average Bandwidth and Average Peak Frequency

**FIGURE 4:**
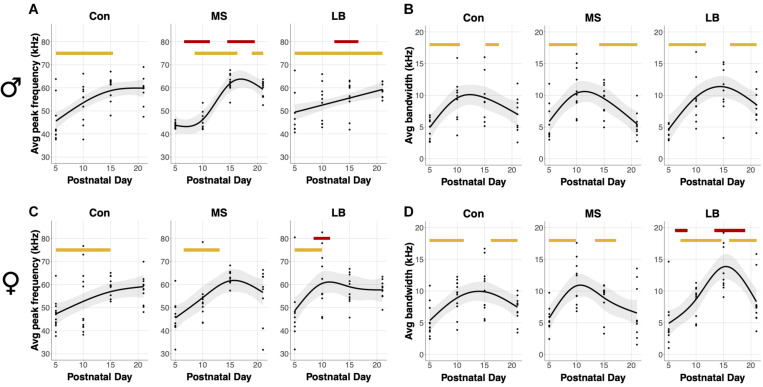
Effects of age and maternal separation (MS) or limited bedding (LB) on peak frequency and bandwidth averaged across all USVs for each subject. Age related increases or decreases are indicated by orange bars. **(A)** In males, peak frequency was decreased by MS P6.61-P11.27 and increased by MS P14.49 and P19.47. LB peak frequency was decreased in males P12.24-16.58. **(B)** There was no effect of MS or LB on developmental changes in bandwidth in males. **(C)** In females, LB increased peak frequency from P8.46-P11.43. **(D)** LB decreased the bandwidth of female calls from P6.21-P8.38 and increased the bandwidth from P13.36-P18.91 *n* = 8–10.

An average bandwidth (maximum frequency – minimum frequency) and average peak frequency across all calls for a given subject were calculated. In males, USVs in all rearing groups increased in average peak frequency as age increased (SST: Con: *F* = 7.396, *p* = 0.001^∗∗^; MS: *F* = 7.521, *p* < 0.001^∗∗∗^; LB: *F* = 6.564, *p* < 0.001^∗∗∗^, Figure 4A). Specifically, age-related increases occurred between P5 and P15.37 in Con, between P8 and P16.26 in MS, and between P5 and P21 in LB. Interestingly, the GAM was reduced to a linear relationship in LB males, as indicated by an effective degrees of freedom (edf) equal to 1. MS peak frequency was lower than Con between P6.61 and P11.27, and greater than Con between P14.49 and P19.47. LB peak frequency was less than Con from P12.24-16.58. Males in all rearing conditions also increased the average bandwidth of calls across development, and no group was significantly different from Con at any age (SST: Con: *F* = 6.609, *p* = 0.002^∗∗^; MS: *F* = 7.678, *p* < 0.001^∗∗∗^; LB: *F* = 6.8.652, *p* < 0.001^∗∗∗^, Figure 4C). Con males increased USV bandwidth from P5-P10.47 and decreased from P15.21-P17.54. Similarly, bandwidth increased in MS males between P5 and P10.07 and decreased from P14.17 until P21. LB USVs increased in bandwidth from P5-P11.75 and decreased from P16.18 until P21.

In females, there was a developmental increase in the average peak frequency of USVs in all groups (SST: Con: *F* = 3.889, *p* = 0.022^∗^; MS: *F* = 4.13, *p* = 0.023^∗^; LB: *F* = 4.89, *p* = 0.006^∗∗^, Figure 4B). This increase occurred from P5 to P14.89 in Con, P6.53 to P13.04 in MS, and P5 to P9.9 in LB-reared animals, and LB peak frequency was greater than Con between P8.46 and P11.43. The average bandwidth of female calls also depended on age in all rearing conditions (SST: Con: *F* = 5.909, *p* = 0.014^∗^; MS: *F* = 6.2, *p* = 0.002^∗^; LB: *F* = 11.848, *p* < 0.001^∗∗∗^, Figure 4D), with Con pups increasing between P5 and P11.19 and decreasing from P16.18 to P21. The model predicted an increase in MS bandwidth between P5 and P9.82 and decrease between P13.37 and P17.14. In LB females, bandwidth increased from P7.17 until P14.57 and decreased from P16.1 until P21. The model predicted that in females, LB bandwidths were, on average, lesser than Con between P6.21 and P8.38, and greater than Con between P13.36 and P18.91.

Additional GAM analyses comparing males and females within rearing conditions demonstrated age-related changes in both sexes in all rearing conditions. In Cons, there were no differences between males and females. In MS, female peak frequency was greater than males between P7.01 and P12.08, but less than males between P18.11 and P20.84. In LB, female peak frequency was greater than males between P8.54 and P13.2.

### Maternal Behavior

#### Entropy Rate

**FIGURE 5:**
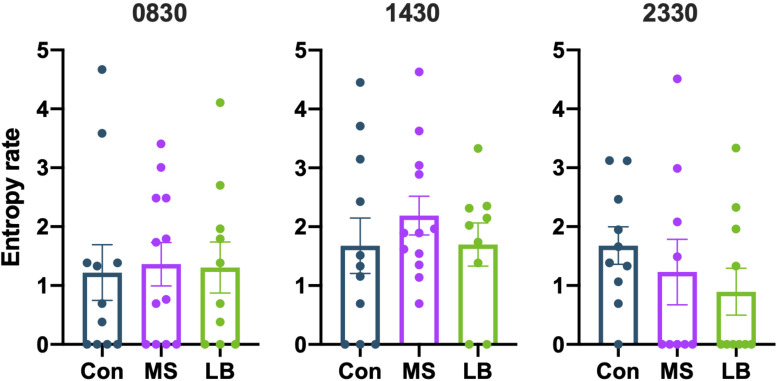
Maternal separation (MS) and limited bedding (LB) did not affect the entropy of maternal behavior sequences at 0830, 1430, or 2330. *n* = 9–12.

To assess the overall predictability of maternal behavior, entropy rates were calculated at 0830, 1430, and 2330. Entropy rates were not observed to be affected by MS or LB at any time point [0830: *F*_(2_,_32)_ = 0.02991, *p* = 0.9706, η*_*p*_*^2^ = 0.002; 1430: *F*_(2,31)_ = 0.5729, *p* = 0.5701, η*_*p*_*^2^ = 0.0038; 2330: *F*_(2,28)_ = 0.8804, *p* = 0.4266, η*_*p*_*^2^ = 0.0634; Figure 5]. To ensure that entropy was not attributable to variability in pup weight, ANCOVAs were conducted for maternal entropy at each of three time points with pup weight at P8 as a covariate (Supplementary Table 3). Pup weight did not covary with maternal entropy scores (*p* > 0.05 at all time points for both males and females).

#### On Nest Duration

**FIGURE 6:**
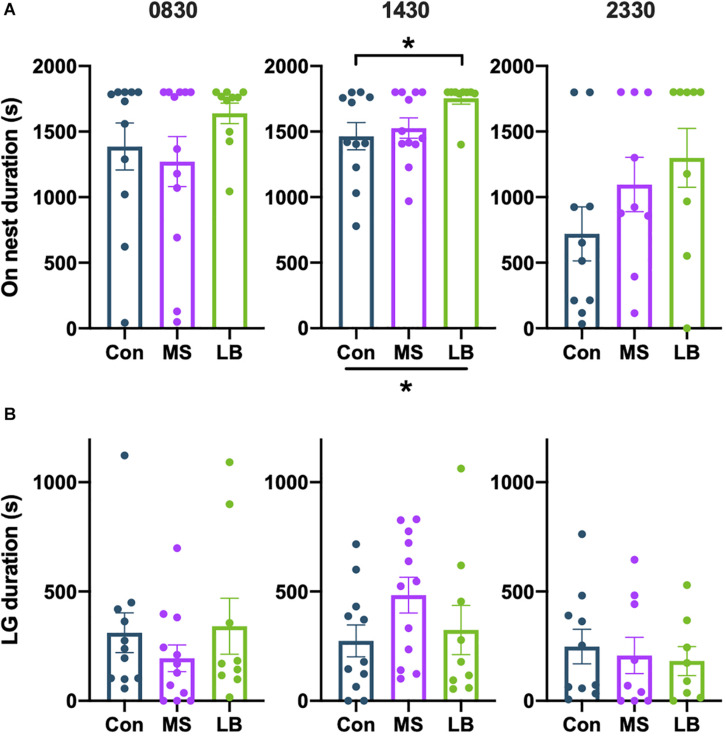
Effects of rearing on **(A)** time spent on the nest and **(B)** time spent licking and grooming (LG). LG was increased in LB dams compared to Con at 1430. *p < 0.05. *n* = 9–12.

There was no effect of rearing on the total time spent on the nest at 0830 [*F*_(2_,_32)_ = 1.281, *p* = 0.2926, η*_*p*_*^2^ = 0.0787] or 2330 [*F*_(2,27)_ = 1.961, *p* = 0.1618, η*_*p*_*^2^ = 0.1356] (Figure 6A). At 1430, data failed the Shapiro-wilk test of normality (*p* = 0.0214), but a Kruskal-Wallis test showed that rearing affected on-nest duration [H_(2)_ = 7.397, *p* = 0.0248, Figure 6A]. *Post-hoc* comparisons reveal that this effect is likely driven by increased time spent on the nest by LB dams compared to Con dams (*p* = 0.0255).

#### Licking and Grooming

There was no observed effect of rearing on the total time spent licking and grooming (LG) at any time point [0830: *F*_(2,31)_ = 0.7399, *p* = 0.4860, η*_*p*_*^2^ = 0.0485; 1430: *F*_(2,31)_ = 1.655, *p* = 0.2087, η*_*p*_*^2^ = 0.1024; 2330: *F*_(2,26)_ = 0.1864, *p* = 0.8311, η*_*p*_*^2^ = 0.0153] (Figure 6B), and no effect on the total number of LG bouts [0830: *F*_(2,29)_ = 1.275, *p* = 0.1952, η*_*p*_*^2^ = 0.0835; 1430: *F*_(2,29)_ = 0.5690, *p* = 0.5723, η*_*p*_*^2^ = 0.0378; 2330: *F*_(2,27)_ = 0.2448, *p* = 0.7847, η*_*p*_*^2^ = 0.0192] (Figure 6B).

#### Nursing

**FIGURE 7:**
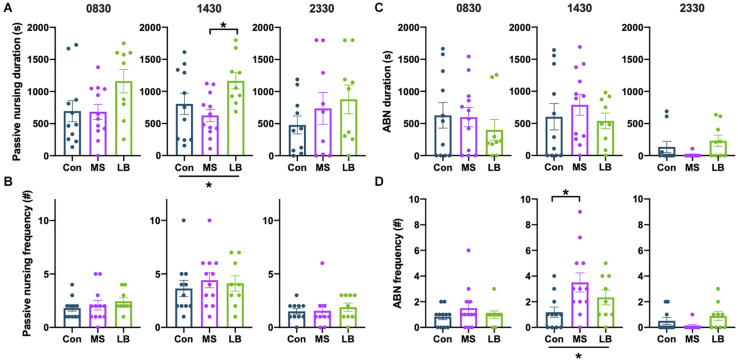
Effects of maternal separation (MS) or limited bedding (LB) on passive nursing and arched-back nursing (ABN) durations **(A,C)** and frequencies **(B,D)** at 0830, 1430, and 2330. At 1430, LB dams spend more time ABN than MS dams, but nursing did not differ at 0830 or 2330. At 1430, MS dams were more frequently initiating ABN bouts than Con dams, but the total duration of ABN did not differ. *p < 0.05. *n* = 9–12.

Arched-back nursing (ABN) and passive nursing bouts were quantified separately. There was a significant effect of rearing on the total time dams spent passively nursing at 1430 [*F*_(2,31)_ = 4.105, *p* = 0.0269, η*_*p*_*^2^ = 0.0629], and *post-hoc* comparisons revealed that this effect was driven by an increase in passive nursing in LB dams compared to MS dams (*p* = 0.0212) (Figure 7A). There were no effects of rearing on the time dams spent passive nursing at 0830 [*F*_(2,31)_ = 2.896, *p* = 0.0713, η*_*p*_*^2^ = 0.1665] or 2330 [*F*_(2,27)_ = 0.9960, *p* = 0.3835, η*_*p*_*^2^ = 0.0738], and no effect on the number of passive nursing bouts [0830: *F*_(2,31)_ = 0.6294, *p* = 0.54, η*_*p*_*^2^ = 0.0416; 1430: *F*_(2,31)_ = 0.3072, *p* = 0.7379, η*_*p*_*^2^ = 0.0207; 2330: *F*_(2,27)_ = 0.2343, *p* = 0.7928, η*_*p*_*^2^ = 0.0184] (Figure 7B).

The total time spent ABN was not affected by rearing condition at any time point [0830: *F*_(2,31)_ = 0.4701, *p* = 0.6296, η*_*p*_*^2^ = 0.0314; 1430: *F*_(2,31)_ = 0.5839, *p* = 0.5641, η*_*p*_*^2^ = 0.0387; 2330: *F*_(2,27)_ = 2.210, *p* = 0.1306, η*_*p*_*^2^ = 0.1503] (Figure 7C). However, rearing affected the number of ABN bouts at 1430 [*F*_(2,31)_ = 3.904, *p* = 0.0315, η*_*p*_*^2^ = 0.2121], but not at 0830 [*F*_(2,31)_ = 0.9739, *p* = 0.3896, η*_*p*_*^2^ = 0.0629] or 2330 [*F*_(2_, _27)_ = 2.090, *p* = 0.1447, η*_*p*_*^2^ = 0.1433] (Figure 7D). This effect was driven by an increase in ABN bouts in MS dams compared to Con (*p* = 0.0241) (Figure 7D).

### Maternal Entropy Predicted Total USVs Emitted by Females at P21

**FIGURE 8:**
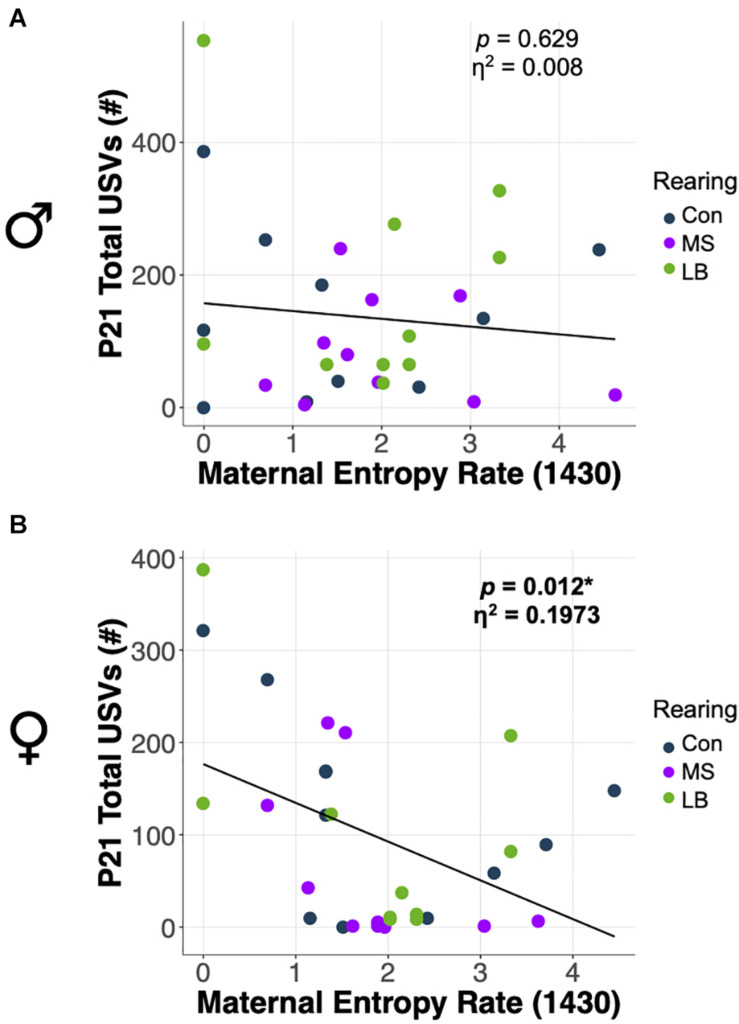
Effects of maternal entropy rate at 1430 on total USV emissions at P21 in **(A)** males and **(B)** females. *p < 0.05, *n* = 10–11.

ANCOVA were conducted to determine whether total USVs at weaning were related to the predictability of postnatal maternal care. Statistics for all ANCOVA can be found in Supplementary Table 4. In females, entropy rate at 1430 was negatively correlated with the number of USVs emitted by pups at P21 (*p* = 0.012; η^2^ = 0.1973) (Figure 8B), and there was no effect of rearing on total USVs after correcting for entropy rate. In males, there was no effect of entropy rate at any time on the total number of USVs emitted (*p* = 0.316; η^2^ = 0.0739) (Figure 8A).

## Discussion

Identifying specific factors of adverse experiences that contribute to later-life consequences will help to generalize findings from rodent studies for use in the human realm. To this end, the current study determined the effects of two adversity models, MS and LB, on USV emissions and patterns of maternal care. We aimed to characterize typical developmental trajectories of infant communication, and the extent to which maternal behavior and adversity impacted USV maturation. Overall, our findings show that MS and LB altered total USV emissions, peak frequency, and four call types in a sex-specific manner, and total USVs at P21 were associated with the predictability of postnatal maternal care. This final finding highlights the impact of a specific aspect of maternal behavior on behavioral development in all rearing conditions.

There are limitations to the study design that should be noted when interpreting the findings. First, pups were recorded in small cups in a water bath at P5 and P10 and in small cages at P15 and P21. Recording in the water bath at P5 and P10 was necessary since reduced core temperature has an effect on USVs that is distinct from the effects of isolation (Hahn and Lavooy, 2005), and this study aimed to test the USV response due to the experience of isolation alone, and not additional environmental stressors. To maintain consistency between the two contexts, home cage bedding was provided in both to limit any novel olfactory cues. Therefore, although the recording protocol attempted to minimize changes between conditions at P5/P10 and P15/P21 while maintaining core body temperature at all times recorded, age-related changes in USV trajectory should be interpreted with this consideration. Another limitation is the difference in the timelines of MS and LB paradigms. The P2-P20 MS timeline was implemented to align with the methods used in previous findings from our lab, while the P2-P14 is more typical for the LB protocol (Walker et al., 2017). Since LB ended on P14 and pup USVs were recorded on P15, changes in USVs seen during this time may be a response to returning to standard rearing cages. For these reasons, we avoided directly comparing the USV development curves of MS and LB, and instead interpret the results in relation to the control group. For maternal behavior, however, both adversity groups had undergone MS or LB for the same amount of time at the time of recording (P8-P9), so direct comparisons are made between Con, MS, and LB. Future work will determine the extent to which MS and LB affect USV trajectories if their timelines are identical.

We report an age-dependent increase in USV emissions during the first half (through P12) of the preweaning period in males and females reared in standard conditions. Heightened USV emissions at P11-P12 temporally aligns with the approximate end of the stress hyporesponsive period (SHRP) in rodents. In rats, the SHRP is the estimated period between P4 and P14 characterized by low basal corticosterone levels and a suppressed neuroendocrine response to stressors (Sapolsky and Meaney, 1986). Our findings align with others who have shown that vocalizations are highest between P6 and P12 (Noirot, 1968; Insel et al., 1986; Brudzynski, 2019). Increases in USVs during this period with maximum emission rates emerging by P15 could therefore indicate maturation of the HPA axis.

In males undergoing MS, USV emissions were increased earlier in the SHRP compared to Con, consistent with a previous report that male pups undergoing MS emitted more USVs than controls at P12 (Kaidbey et al., 2019). Prolonged separation periods from the dam during the SHRP invokes corticosterone release in otherwise quiescent pups (Levine, 2001); therefore enhanced vocalization rates at a younger age while undergoing MS may point to a change in typical development of HPA axis responsivity (Branchi et al., 2001; Hofer et al., 2001; Levine, 2001; Dirks et al., 2002). Notably, we found this response to MS to be specific to males. No effect of MS was seen in females, while USVs were initially suppressed by LB until P10. After approximately P14, LB females emitted more USVs than Con. The initial suppression of USVs in females may be due to sex differences in typical HPA axis maturation (Yoshimura et al., 2003) and the curvilinear fashion by which corticotropin releasing factor (CRF) modulates USVs; CRF at moderate levels dose-dependently invoke USVs, but USVs are eventually quieted with high levels of CRF (Dirks et al., 2002). Sex differences in maternal care directed at individual pups may also contribute to differences in USVs (Richmond and Sachs, 1983). Although not measured here, females have been shown to receive higher levels of maternal behaviors resembling abuse when reared in adversity models, while males receive more tactile stimulation and general contact (Keller et al., 2019). Decreased USV emissions in LB females may represent an adaptive avoidance strategy if maternal contact is abusive or painful (Moriceau et al., 2009); however, deconstructing the specific factors of LB that lead to female-specific effects will require analysis of maternal care on individual pups.

Peak frequency, bandwidth, and sonographic structure of USVs were also altered depending on sex and adversity type. There was a developmental increase in average peak frequency beginning at P5 and continuing until approximately P15. In males, MS initially hindered this increase, but later, between P15 and P20, peak frequency was elevated compared to Con. Comparing males to females within each rearing condition showed that male peak frequency was higher than females during this period. Kaidbey et al. (2019) found a similar pattern of higher USV frequency in MS pups during both an initial and secondary separation from the dam. In females, we found that peak frequency was elevated by LB, but not MS, and this elevation occurred around P10. Again, this demonstrates an effect of MS in males and LB in females.

Since the frequency of a vocalization is influenced by its sonographic structure (Ehret, 2005), we also quantified USVs by proportions of call types. Identifying developmental changes in particular call types could reveal acoustic characteristics that are functionally relevant at particular ages (Brudzynski et al., 1999). We found that the proportion of specific call types emitted by Con males and females fluctuated with age. Brudzynski et al. (1999) argues that developmental profiles of specific USV waveforms in the rat could indicate adaptive significance for communication and facilitation of maternal care. The occurrence of complex calls, characterized by recurrent frequency modulation, increased over development and composed more than 50% of all call types by P21. Complex calls could thereby signify a more mature vocal repertoire.

Reported effects of ELA on maternal behavior have been mixed and seem to largely depend on the type of adversity used, measures assessed, and particular experimental procedures which widely vary between labs (Orso et al., 2019). Recently, more attention has been given to signs of fragmented or erratic maternal behavior, especially in LB paradigms, which are argued to induce unpredictable care patterns (Molet et al., 2016). We did not observe effects of MS or LB on the predictability of maternal behavioral sequences as measured by entropy rates, yet it should be noted that others have recorded maternal behavior over 60 or 180-min periods, while we only observed 30-min windows. Further, we did not code off-nest behaviors—aside from self-grooming. Including more diverse behaviors in the analysis and extending the observation period could produce results more sensitive to potential behavioral changes and should be considered for future studies. Although we did not find group differences in entropy rate, we hypothesized that entropy rate variability is linked to pup USV emissions. In fact, we show that entropy at 1430 negatively correlated with total USVs in females at P21. This suggests that maternal entropy rate could be a common factor of the rearing environment, regardless of adversity condition, that influences USVs at weaning age. To test the validity of this correlation between USVs and maternal care, experiments directly testing the maternal response to USVs must be performed. An important addition to this line of work will be determining whether differences in USVs due to rearing condition or individual differences impact maternal retrieval behavior.

Previous investigations in both humans and rodents have found certain features of distress vocalizations to be functionally significant in parent-infant relationships (Blumberg and Sokoloff, 2001; Zeskind et al., 2011). In humans, spectral features of infant cries are associated with developmental disturbances and have been used as an early identifier of medical conditions and developmental disorders (LaGasse et al., 2005; Zeskind et al., 2011; Sheinkopf et al., 2012). For example, shifts to higher fundamental frequencies in infant crying is associated with prenatal cocaine exposure (Zeskind et al., 2011) and are more pronounced in infants later diagnosed with autism spectrum disorder (Sheinkopf et al., 2012). Additionally, infants born prematurely are at an increased risk for vocal and cognitive impairments characteristic of developmental disorders, which may be linked to prolonged periods of maternal separation in the neonatal intensive care unit (Beebe et al., 2018). Family Nurture Intervention programs curtail these deficits by increasing quality mother-infant interactions through scent exchange, direct touch, and vocal communication from mother to infant (Welch et al., 2015). While rodent vocalizations are not analogous to human cries, comparing similar features of species-specific distress signals can validate these models for translational approaches to studying interventions for neurodevelopmental disorders. Indeed, the ability for preclinical research to implement characterizations of socially relevant communication (i.e., USVs) may provide critical translational insight into the developmental dynamics of communication, which are often found to be disrupted in developmental and psychiatric disorders.

Although rodent models of ELA are widely used, discussions of potential differences in their effects on the offspring experience are often ignored. Overall, this study demonstrates that infant vocalizations in rats follow a developmental pattern that can be referenced to decipher impacts of the early environment on maturation. Two different adversity paradigms each affected maternal behavior and pup communication strategies, and predictable maternal behavior was a common factor influencing female pup development. Assessing ultrasonic communication in rodents can serve as a translational tool for researchers to better characterize early neurobehavioral disturbances, contributing to our understanding of the etiology of psychiatric vulnerabilities in humans exposed to ELA.

## Data Availability Statement

The raw data supporting the conclusions of this article will be made available by the authors, without undue reservation.

## Ethics Statement

The animal study was reviewed and approved by the Northeastern Institutional Animal Care and Use Committee.

## Author Contributions

LG, JH, and HB conceptualized and designed the experiments. LG, AV, and JLH collected data. LG performed statistical analysis and wrote the original draft. AV, JLH, JH, and HB edited the original draft. All authors contributed to the article and approved the submitted version.

## Conflict of Interest

The authors declare that the research was conducted in the absence of any commercial or financial relationships that could be construed as a potential conflict of interest.
