# Supplementary material for: Trajectories of Mother-Infant Communication: An Experiential Measure of the Impacts of Early Life Adversity
Source: Front Hum Neurosci. 2021 Feb 17;15:632702. doi: 10.3389/fnhum.2021.632702 (PMC7928287; doi:10.3389/fnhum.2021.632702)
Supplement: Supplementary file 1 [file Table_1.DOCX]

Supplementary Table 1. Summary statistics of USV acoustic properties analyzed by generalized additive models comparing males and females within each rearing condition. Effective degrees of freedom (edf) indicates the degree of non-linearity of the age-outcome relationship (edf = 1 for linear relationships). Periods of age-related change based on derivates of GAM models are denoted by (+) for increases and (-) for decreases. Significant differences between males and females are based on differences in GAM predictions and 95% confidence intervals. Measures with effect of age and sex. *p < 0.05, **p < 0.01, ***p < 0.001. n = 9-10.

|  |  |  | | edf | F | p |  | Periods of age-related changes | Age periods significantly different (males vs. females) |
| --- | --- | --- | --- | --- | --- | --- | --- | --- | --- |
| Total USVs (#) | Con | Males | | 2.232 | 4.382 | 0.00661 | ** | 5-11.59 (+), 16.58-21 (-) |  |
|  |  | Females | | 2.065 | 3.687 | 2.27E-02 | * | 5-10.71 (+) |  |
|  | MS | Males | | 2.647 | 8.373 | 3.66E-04 | *** | 5-9.74 (+), 13.12-21 (-) |  |
|  |  | Females | 1.995 | | 3.072 | 0.043713 | * | 15.05-21 (-) |  |
|  | LB | Males | | 2.782 | 4.65 | 0.00365 | ** | 8.94-14.97 (+), 18.35-21 (-) |  |
|  |  | Female | | 2.919 | 9.661 | 1.20E-05 | *** | 8.78-15.21 (+), 16.34-21 (-) |  |
| ***Avg Peak Frequency (kHz)*** | Con | Male | | 1.788 | 8.764 | 0.000263 | *** | 5-14.89 (+) |  |
|  |  | Female | | 1.737 | 7.134 | 1.14E-03 | ** | 5-14.97 (+) |  |
|  | MS | Male | | 2.909 | 19.73 | 4.89E-10 | *** | 8.54-16.26 (+), 19.23-21 (-) | 7.01-12.08 (F > M), 18.11-20.84 (F < M) |
|  |  | Female | | 2.7 | 11.41 | 0.00000217 | *** | 5.72-14.57 (+), 17.06-21 (-) |  |
|  | LB | Male | | 1 | 6.496 | 0.0129 | * | 5-21 (+) | 8.54-13.2 (F > M) |
|  |  | Female | | 2.634 | 4.586 | 1.42E-02 | * | 5-9.98 (+) |  |
| Avg Bandwidth (kHz) | Con | Male | | 2.467 | 7.599 | 0.000905 | *** | 5-10.47 (+), 14.97-17.94 (-) |  |
|  |  | Female | | 2.208 | 6.848 | 8.39E-04 | *** | 5-11.27 (+), 15.85-21 (-) |  |
|  | MS | Male | | 2.528 | 8.653 | 3.52E-04 | *** | 5-10.15 (+), 14.01-21 (-) |  |
|  |  | Female | | 2.766 | 7.026 | 0.001147 | ** | 5-9.82 (+), 13.04-17.22 (-) |  |
|  | LB | Male | | 2.194 | 6.805 | 0.000977 | *** | 5-11.59 (+), 17.3-19.55 (-) |  |
|  |  | Female | | 2.785 | 9.112 | 2.15E-05 | *** | 7.33-14.33 (+), 16.18-21 (-) |  |

Supplementary Table 2: Effect of rearing and pup weight at P20 on P21 USVs

| P21 USVs |  | Male | | Female | |
| --- | --- | --- | --- | --- | --- |
|  | **Effect** | F | *p* | F | *p* |
|  | P20 weight | 1.134 | 0.298 | 0.799 | 0.380 |
|  | Rearing | 0.947 | 0.402 | 0.494 | 0.616 |
|  | Rearing x P20 weight | 0.282 | 0.757 | 0.185 | 0.832 |

Supplementary Table 3: Effect of rearing and pup P9 weight on maternal entropy rate

| 0830 entropy rate |  | Male | | Female | |
| --- | --- | --- | --- | --- | --- |
|  | **Effect** | F | *p* | F | *p* |
|  | P9 weight | 2.565 | 0.122 | 0.969 | 0.334 |
|  | Rearing | 0.417 | 0.664 | 0.219 | 0.805 |
|  | Rearing x P9 weight | 0.617 | 0.548 | 0.447 | 0.644 |

| 1430 entropy rate |  | Male | | Female | |
| --- | --- | --- | --- | --- | --- |
|  | **Effect** | F | *p* | F | *p* |
|  | P9 weight | 0.149 | 0.703 | 0.067 | 0.798 |
|  | Rearing | 0.531 | 0.595 | 0.011 | 0.989 |
|  | Rearing x P9 weight | 1.24 | 0.307 | 0.573 | 0.571 |

| 2330 entropy rate |  | Male | | Female | |
| --- | --- | --- | --- | --- | --- |
|  | **Effect** | F | *p* | F | *p* |
|  | P9 weight | 1.406 | 0.248 | 1.187 | 0.287 |
|  | Rearing | 1.442 | 0.258 | 1.685 | 0.208 |
|  | Rearing x P9 weight | 0.6 | 0.558 | 1.022 | 0.375 |

Supplementary Table 4: Effects of rearing on total USVs adjusting for maternal entropy rate at 0830, 1430, or 2330. **Adjusted p < 0.0127.**

*Analyses failing assumptions of normality or homogeneity of variances are italicized*

Covariate: 0830 Entropy Rate

|  |  | P5 | | | | | P10 | | | | | P15 | | | | | P21 | | | | |
| --- | --- | --- | --- | --- | --- | --- | --- | --- | --- | --- | --- | --- | --- | --- | --- | --- | --- | --- | --- | --- | --- |
|  |  | Male | | Female | | Male | | | Female | | Male | | | Female | | Male | | | Female | |  |
|  | **Effect** | F | *p* | F | *p* | F | | *p* | F | *p* | F | | *p* | F | *p* | F | | *p* | F | *p* |  |
| Total USVs | Entropy | 1.232 | 0.278 | *0.016* | *0.9* | *1.992* | | *0.17* | *0.818* | *0.374* | 0.436 | | 0.515 | 0.213 | 0.648 | 1.942 | | 0.175 | 2.816 | 0.105 |  |
|  | Rearing | 3.046 | 0.065 | *5.303* | ***0.011*** | *10.641* | | ***0.00042*** | *4.206* | *0.026* | 0.027 | | 0.973 | 1.139 | 0.335 | 1.687 | | 0.205 | 1.173 | 0.325 |  |

Covariate: 1430 Entropy Rate

|  |  | P5 | | | | | P10 | | | | | P15 | | | | | P21 | | | | |
| --- | --- | --- | --- | --- | --- | --- | --- | --- | --- | --- | --- | --- | --- | --- | --- | --- | --- | --- | --- | --- | --- |
|  |  | Male | | Female | | Male | | | Female | | Male | | | Female | | Male | | | Female | |  |
|  | **Effect** | F | *p* | F | *p* | F | | *p* | F | *p* | F | | *p* | F | *p* | F | | *p* | F | *p* |  |
| Total USVs | Entropy | *0.002* | *0.967* | *1.241* | *0.275* | *0.081* | | *0.779* | *1.5* | *0.231* | 1.661 | | 0.209 | 3.109 | 0.089 | 0.24 | | 0.628 | 7.17 | **0.012*** |  |
|  | Rearing | 2.551 | 0.098 | *5.531* | ***0.01*** | *10.324* | | ***0.0005*** | *4.616* | *0.019* | 0.051 | | 0.951 | 1.204 | 0.316 | 1.303 | | 0.289 | 1.147 | 0.333 |  |

Covariate: 2330 Entropy Rate

|  |  | P5 | | | | | P10 | | | | | P15 | | | | | P21 | | | | |
| --- | --- | --- | --- | --- | --- | --- | --- | --- | --- | --- | --- | --- | --- | --- | --- | --- | --- | --- | --- | --- | --- |
|  |  | Male | | Female | | Male | | | Female | | Male | | | Female | | Male | | | Female | |  |
|  | **Effect** | F | *p* | F | *p* | F | | *p* | F | *p* | F | | *p* | F | *p* | F | | *p* | F | *p* |  |
| Total USVs | Entropy | *3.161* | *0.089* | *0.023* | *0.881* | *0.758* | | *0.393* | 0.594 | 0.448 | 0.336 | | 0.567 | 2.63 | 0.117 | 0.222 | | 0.642 | 0.099 | 0.756 |  |
|  | Rearing | 2.644 | 0.093 | *6.282* | ***0.006*** | *11.416* | | ***0.00032*** | 2.839 | 0.077 | 0.064 | | 0.938 | 1.979 | 0.159 | 0.966 | | 0.395 | 0.589 | 0.562 |  |
